# The Mediating Role of Emotion Dysregulation in the Association Between Impulsivity and Severity of Substance Dependence

**DOI:** 10.1002/cpp.70250

**Published:** 2026-03-09

**Authors:** Giovanni Mansueto, Sara Palmieri, Ana V. Nikčević, Gabriele Caselli, Marcantonio M. Spada

**Affiliations:** ^1^ Department of Health Sciences University of Florence Florence Italy; ^2^ School of Applied Sciences London South Bank University London UK; ^3^ Department of Psychology Sigmund Freud University Milan Italy; ^4^ Cognitive Psychotherapy School and Research Center Studi Cognitivi Milan Italy; ^5^ Department of Psychology, School of Law, Social and Behavioural Sciences Kingston University Kingston UK

**Keywords:** dependence, emotion dysregulation, emotion regulation, impulsivity, substance

## Abstract

Substance use disorders (SUDs) are among the leading causes of morbidity and mortality globally. Identification of aetiological factors related to the severity of substance use can offer targets for therapeutic interventions. Role of impulsivity and emotion dysregulation in SUDs is well established. Based on the evidence that the adverse effects of impulsivity on behavioural and mental health outcomes can be mediated by emotion dysregulation, this study explored the possible mediating role of emotion dysregulation in the association between impulsivity and the severity of substance dependence. One hundred treatment‐seeking substance users (monosubstance users and polysubstance users) were enrolled. Impulsivity, emotion dysregulation and severity of substance dependence were assessed using the Barratt Impulsiveness Scale‐11, the Difficulties Emotion Regulation Scale and the Severity Dependence Scale, respectively. Correlation, mediation and 95% bias‐corrected and accelerated (BCa CI) bootstrapped analyses were run. Emotion dysregulation was found to mediate the association between attention impulsivity (indirect effect [BCa CI]: 0.22 [0.11–0.36]), motor impulsivity (indirect effect [BCa CI]: 0.15 [0.08–0.24]), non‐planning impulsivity (indirect effect [BCa CI]: 0.11 [0.04–0.18]) and severity of substance dependence. The direct effect of different facets of impulsivity on dependence severity was not significant after accounting for emotion dysregulation. Impulsivity was found to have a significant indirect effect on the severity of substance use through emotion dysregulation. Among substance users, emotion dysregulation may be involved in the association between impulsivity and severity of substance dependence. Emotion dysregulation could be considered a possible therapeutic target to reduce the severity of substance dependence among substance users with impulsive tendencies.

## Summary

- Assessing impulsivity and emotion dysregulation in substance users may enable clinicians to design more tailored interventions to reduce the severity of substance dependence.
- Substance users who exhibit impulsive tendencies may experience greater difficulties in regulating their own emotions.
- In substance users with impulsivity tendencies, emotion dysregulation may exacerbate the severity of substance dependence.
- Emotion dysregulation could serve as a therapeutic target to reduce the severity of substance dependence among substance users with impulsive tendencies.

## 1 Introduction

Substance use disorders (SUDs) are defined as patterns of substance use that cause damage to physical or mental health or lead to clinically significant functional impairment or distress (Volkow and Blanco 2023). Among SUDs, the prevalence is highest for nicotine use disorder and alcohol use disorder, followed by opioid use disorder and cannabis use disorder (Volkow and Blanco 2023). The impact of SUDs on societies as it relates to health and mortality, economics and crime is profound, and it appears to be worsening (Volkow and Blanco 2023). SUDs are among the leading causes of morbidity and mortality globally (Connery et al. 2020; Volkow and Blanco 2023).

Exploration of etiological factors underlying the severity of substance dependence is important as it may allow for the identification of modifiable factors that could be targeted in clinical interventions (Connery et al. 2020; Volkow and Blanco 2023). Among various such factors, impulsivity and emotion dysregulation have been found to be related to the severity of substance dependence in several studies (Loree et al. 2015; DeVito et al. 2020; Stellern et al. 2023; Weiss et al. 2022).

### 1.1 Impulsivity and Severity of Substance Dependence

Impulsivity is considered a multidimensional concept (Sharma et al. 2014; Whiteside and Lynam 2001), defined as a tendency to react in a rapid and unplanned way to internal or external stimuli, with little thought for the negative consequences of one's actions (Barratt and Patton 1983; Stanford et al. 2009). Impulsivity is typically measured via self‐report personality questionnaires, and behaviourally, using various experimental tasks (Goodwin et al. 2023). One of the most commonly used self‐report measures of impulsivity is the Barratt Impulsivity Scale (BIS‐11) (Patton et al. 1995), which has been described as the ‘gold standard’ assessment tool of trait impulsivity (Stanford et al. 2009). According to the BIS‐11 three dimensions of impulsivity have been identified, that is, attentional impulsiveness that is inability to focus one's attention and quick decision making, motor impulsiveness involving acting without thinking and nonplanning impulsiveness, that is, inability to plan for present/future (Barratt 1985).

It has been suggested that substance users report higher levels of self‐reported impulsivity relative to controls (DeVito et al. 2020; Stanford et al. 2009). Elevated impulsivity scores have been found to be associated with the greater number of cigarettes smoked (Ryan et al. 2013; Stanford et al. 2009), problematic alcohol (Bernstein et al. 2015; Messina et al. 2014; Perry and Carroll 2008) and significant escalation of problematic drug use (DeVito et al. 2020; Messina et al. 2014; Perry and Carroll 2008; Stanford et al. 2009; Wagner et al. 2022). Overall, cumulative evidence to date suggests that higher impulsivity is associated with the greater substance dependence (Loree et al. 2015; DeVito et al. 2020). Moreover, there is evidence that among substance users elevated impulsivity scores are also associated with a higher risk of substance use relapse and worse treatment outcome (DeVito et al. 2020; Loree et al. 2015; Makarenko et al. 2025).

### 1.2 Emotion Dysregulation and Severity of Substance Dependence

Emotion dysregulation is a multifaceted construct involving maladaptive ways of responding to one's emotions, in an attempt to regulate one's emotional states (Gratz and Roemer 2004). Emotion dysregulation may be assessed through self‐report questionnaires and, among these, one of the most commonly used self‐report measures of it is the Difficulties in Emotion Regulation Scale (DERS) (Gratz and Roemer 2004). According to the DERS, a comprehensive conceptualization of emotion dysregulation encompasses multiple maladaptive ways of responding to one's emotions including (a) lack of emotional awareness, clarity and acceptance; (b) behavioural dyscontrol in the context of intense emotions; (c) unwillingness to pursue meaningful activities in the context of emotional distress; and (d) inflexible use of adaptive strategies to modulate the intensity and/or duration of emotional experiences (Gratz and Roemer 2004).

It has been suggested that substance users report higher levels of emotion dysregulation relative to controls (Mansueto, Palmieri, et al. 2024; Stellern et al. 2023; Weiss et al. 2022). Participants with SUDs, compared with those without SUDs, were found to have more difficulties in concentrating and accomplishing tasks when experiencing negative emotions, in accessing strategies to effectively regulate emotions, in keeping control of one's behaviour when experiencing negative emotions, in being aware of personal emotional responses and in understanding experienced emotions (Mansueto, Palmieri, et al. 2024; Stellern et al. 2023; Garke et al. 2021). Higher levels of emotion dysregulation have been found to be associated with a higher frequency of substance use, polysubstance use and greater substance dependence severity (Stellern et al. 2023; Weiss et al. 2022). Furthermore, there is evidence that among substance users, emotion dysregulation is also associated with a higher risk of substance use relapse and worse treatment outcome (Berking et al. 2011; Clarke et al. 2020; Stellern et al. 2023).

### 1.3 Impulsivity and Severity of Substance Dependence: A Possible Indirect Effect of Emotion Dysregulation

Although the independent contribution of both impulsivity and emotion dysregulation on the severity of substance dependence is well documented (Loree et al. 2015; DeVito et al. 2020; Stellern et al. 2023; Weiss et al. 2022), less is known about their reciprocal roles in the pathogenies of the severity of substance dependence (Garofalo and Velotti 2015). This may be due to the partial overlapping of these two constructs (Schreiber et al. 2012). Neuroscientific evidence suggests that this overlap may be attributable to a shared underlying neurobiological substrate that underpins both impulsivity and emotion dysregulation. There is evidence that the prefrontal cortex and the amydgala both may play key roles in emotion regulation, as well in impulsivity, decision making, risk‐taking, motor control and reasoning (e.g., Bechara et al. 2000; Fecteau et al. 2007; Hinvest et al. 2011; Kim and Lee 2010; Krawczyk et al. 2011; Manes et al. 2002; Ochsner and Gross 2005; Ray and Zald 2012; Spinella 2004; Torresgrossa et al. 2008; Xie et al. 2011; Zeeb et al. 2010; Zeeb and Winstanley 2011). Evidence from psychological research suggests a vicious circle between impulsivity and emotion dysregulation, whereby they mutually reinforce each other (e.g., Garofalo, Velotti, and Zavattini 2018; Schreiber et al. 2012; Selby et al. 2008).

To untangle this complex relationship, the Self‐Regulation Deficit perspective suggests that prioritizing affect regulation may contribute to destructive patterns of failed impulse control (Baumeister and Heatherton 1996). In line with this framework, some authors have purported that impulsivity may have an indirect effect on behavioral and mental health outcomes through emotion dysregulation (d'Acremont and Van der Linden 2007; Estévez et al. 2024; Lazuras et al. 2019; Liau et al. 2015). Supporting this hypothesis, empirical evidence indicates that impulsivity may have an indirect effect on health outcomes such as the severity of eating disorder psychopathology (Estévez et al. 2024), severity of depressive psychopathology (d'Acremont and Van der Linden 2007) and pathological video gaming (Liau et al. 2015), via the use of maladaptive emotion regulation strategies.

Based on this evidence, it could be expected that, also among subjects with SUD, impulsivity may have an indirect effect on the severity of substance use trough emotion dysregulation. A brief overview of the literature underpinning the delineation of this hypothesis is presented below. As pointed out by the Self‐Regulatory Executive Function theory (Wells and Matthews 1996) individuals with SUD with impulsive tendencies are likely to engage in maladaptive forms of mental control to regulate their aversive internal states, which, in turn, hinder effective emotion regulation (Mansueto et al. 2022; Mansueto, Caselli, et al. 2024; Mansueto, Jarach, et al. 2024; Sheppes et al. 2015; Spada et al. 2015). In line with this framework, research shown that individuals with SUD who exhibit higher levels of impulsivity (including attentional impulsiveness, motor impulsiveness and nonplanning impulsiveness) also tend to experience greater levels of emotion dysregulation (Garofalo and Velotti 2015; Jakubczyk et al. 2018; Okasha et al. 2021; Russell et al. 2017; Thrap et al. 2013). From the clinical point of view, this means that individuals with SUD who exhibit impulsive tendencies often rely on maladaptive emotion regulation strategies, which instead of alleviating distressing emotions, tend to amplify them (Selby et al. 2008). Consequently, as proposed by several theoretical models (e.g., the Self‐Regulation Deficit perspective; Baumeister and Heatherton 1996; the ‘negative/positive reinforcement model’; Baker et al. 2004; Weiss et al. 2022; the Emotional Cascade Model; Selby et al. 2008), it could be expected that individuals with SUD and impulsivity tendencies would be more likely to engage in substance use due to their reliance on maladaptive emotion regulation strategies, which, in turn, may increase the likelihood of using substances as a means to regulate their distressing emotions.

### 1.4 Aims

Within this framework, the current study aimed to extend our understanding of the relationship between impulsivity, emotion dysregulation and substance dependence severity among a sample of substance users (i.e., monosubstance users and polysubstance users) treatment seekers. We aimed to explore whether impulsivity may have an indirect effect on the severity substance dependence via the use of maladaptive emotion regulation strategies. To date no studies have examined this relationship in subjects with SUD. Understanding these relationships would offer insight into whether, among substance users with impulsive tendencies, emotion dysregulation is a suitable therapeutic target towards the reduction of the severity of substance dependence. The following hypotheses were put forward: (1) attentional impulsiveness may have an indirect effect on the severity substance dependence via emotion dysregulation, (2) motor impulsiveness may have an indirect effect on the severity substance dependence via emotion dysregulation and (3) non‐planning impulsiveness may have an indirect effect on the severity substance dependence via emotion dysregulation.

## 2 Method

### 2.1 Participants and Procedure

This was an observational, cross‐sectional study. One hundred substance users seeking treatment for substance use were consecutively recruited from SUD treatment facilities of North Italian Regions (Lombardy and Veneto) (Mansueto, Jarach, et al. 2024). Inclusion criteria were (a) 18 years of age or above, (b) able to provide informed consent, (c) able to complete the assessment protocol and (d) dependency of one or more substances. Participants were not abstinent and were still actively using substances. Participants were in a state of full sobriety when completing the questionnaires. The only exclusion criterion was the evidence of cognitive deficits or problems affecting the ability of reading, understanding and following the study assessment process. A total of 100 participants were enrolled of whom 38 (38%) were females and 62 (62%) were males. The mean age was 45.46 years (SD = 12.21), and the mean education was 10.73 years (SD = 3.29). Out of 100 participants, 58 (58%) were employed and the remaining 42 (42%) were unemployed.

Regarding substance use, 26 (26%) participants reported to use only one substance (i.e., monosubstance users) and 74 (74%) reported to use more than one substance (i.e., polysubstance users). In the whole sample nicotine had the highest rate of use (*n* = 75, 75%), followed by alcohol (*n* = 60, 60%), cocaine (*n* = 27, 27%), heroin (*n* = 13, 13%), cannabis (*n* = 4, 4%), methadone (*n* = 3, 3%), benzodiazepines (*n* = 8, 8%) and ketamine (*n* = 1, 1%).

Socio‐demographic and clinical information were collected via a set of interview‐based screening questions already used in the past (Mansueto et al. 2016, 2022). Ethics approval for the study was obtained from the ethics committee of Sigmund Freud University, Milan, Italy (n° YCJVRADXBIUQ@C89682). All procedures contributing to this work complied with the ethical standards of the relevant national and institutional committees on human experimentation and with the Helsinki Declaration of 1975, as revised in 2008. All participants provided a signed informed consent.

### 2.2 Measures

Barratt Impulsiveness Scale‐11 (BIS‐11) (Patton et al. 1995) is a 30‐item self‐report measure assessing impulsivity (Patton et al. 1995). The BIS‐11 is composed by three subscales: (a) attentional impulsivity, (b) motor impulsivity and (c) nonplanning impulsivity. The items are rated on a 4‐point Likert scale (from 1 = *rarely/never* to 4 = *almost always/always*). Higher scores indicate greater levels of impulsivity. The BIS‐11 has been shown to possess good psychometric properties (Fossati et al. 2001; Patton et al. 1995; Stanford et al. 2009; Vasconcelos et al. 2012). In the present study, the BIS‐11 showed a good internal consistency (BIS‐11 global score Cronbach's alpha = 0.65; BIS‐11 attentional impulsivity subscale Cronbach's alpha = 0.63; BIS‐11 motor impulsivity subscale Cronbach's alpha = 0.61; BIS‐11 non‐planning impulsivity subscale Cronbach's alpha = 0.59).

Difficulties Emotion Regulation Scale (DERS) (Gratz and Roemer 2004) is a 36‐item self‐report measure assessing difficulties in emotion regulation. The DERS is composed of six subscales: (a) non‐acceptance of emotional responses, (b) difficulties engaging in goal‐directed behaviour, (c) impulse control difficulties, (d) lack of emotional awareness, (e) limited access to emotion regulation Strategies and (f) Lack of Emotional Clarity. The items are rated on a 5‐point Likert scale (from 1 = *almost never* to 5 = *almost always*). For the purpose of this study, we considered the DERS total score where higher scores indicate greater levels of emotion dysregulation (Gratz and Roemer 2004). The DERS has been shown to possess good psychometric properties (Giromini et al. 2012; Gratz and Roemer 2004; Sighinolfi et al. 2010). In the present study, the DERS showed a good internal consistency (Cronbach's alpha = 0.90).

Severity Dependence Scale (SDS) (Gossop et al. 1995, 1997) is a five‐item scale evaluating the severity of dependence upon a range of substances such as drug, illicit drug (Bruno et al. 2009; Gossop et al. 1995, 1997), alcohol (Gossop et al. 2002; Lawrinson et al. 2007) and nicotine (Grassi et al. 2014). The SDS has five items: ‘Did you think that your [named substance] use was out of control?’ (Item 1), ‘Did you prospect of not taking any [named substance] make you anxious or worried?’ (Item 2), ‘Did you worry about your [named substance] use?’ (Item 3), ‘Did you wish you could stop using your [named substance]?’ (Item 4) and ‘How difficult did you find it to stop, or go without [named of substance]?’ (Item 5). All items are scored on a 4‐point scale. Items 1–4 are scored as 0 = *never*; 1 = *sometimes*; 2 = *often*; 3 = *always*, whereas Item 5 is scored as 0 = *not difficult*; 1 = *quite difficult*; 2 = *very difficult*; 3 = *impossible* (Gossop et al. 1995, 1997). A total SDS score can be obtained by addition of scores for all items. Higher scores indicate a greater degree of dependence. Regarding polysubstance users, the SDS score refers to the overall dependence severity across substances. The SDS has been shown to possess good psychometric properties (Grassi et al. 2014; Gossop et al. 1995, 1997). In the present study, the SDS showed a good internal consistency (Cronbach's alpha = 0.73).

### 2.3 Statistical Analyses

Descriptive analyses were calculated. Skewness and kurtosis were assessed and were considered adequate for a linear model of analysis in a range of ±2 (Gravetter and Wallnau 2016).

Correlation analyses were used to evaluate the association between impulsivity, emotion dysregulation and substance dependence severity. To examine whether the impulsivity may have an indirect effect on the severity of substance dependence via emotion dysregulation, mediation models were run (Hayes and Rockwood 2017). To do this, we conducted simple mediation models using impulsivity (i.e., BIS‐11 total score ad subscales scores) as the independent variable (X), emotion dysregulation (i.e., DERS total score) as the mediator variable (M) and substance dependence severity (i.e., SDS total score) as dependent variable (Y). The three BIS‐11 subscales score (i.e., BIS‐11 attentional impulsivity, BIS‐11 motor impulsivity, BIS‐11 non‐planning impulsivity) were used as separate independent variables in distinct mediation models. Multicollinearity statistics, that is, tolerance index (TI) and variance inflation factor (VIF), were assessed and were considered adequate if TI > 0.2 and VIF < 5 (Barbaranelli and D'Olimpio 2006; Bowerman and O'Connell 1990; Field 2018; Hair et al. 1998). A bootstrapping procedure with *n* = 5000 bootstrap resamples was used to assess indirect effects (Efron and Tibshirani 1994; Ledermann et al. 2025; Hayes and Rockwood 2017; Kenny et al. 1998; MacKinnon et al. 2002; Preacher and Hayes 2004). An indirect effect was considered significant if the 95% bias‐corrected and accelerated bootstrapped confidence interval (BCa CI) excluded zero. In order to reduce potential interpretation bias, mediation analyses were adjusted for age and sex (Stellern et al. 2023; Weiss et al. 2022). Mediation analyses were performed using the Hayes PROCESS Macromodel 4 (Hayes 2013; Preacher and Hayes 2004).

The two‐sided significance level was set at *p* < 0.05. Analyses were performed with SPSS, Version 29 (SPSS Inc.).

## 3 Results

Table 1 shows the means and standard deviations, ranges, skewness and kurtosis for all the study variables. All variables had skewness and kurtosis in the range of acceptability. Correlation analyses are reported in Table 1, showing that among substance users, impulsivity was positively associated with higher emotion dysregulation and with higher severity of substance dependence. Furthermore, emotion dysregulation was positively associated with higher severity of substance dependence.

**TABLE 1:** Means, standard deviations, ranges and intercorrelations among study variables.

		*M*	SD	Skeweness	Kurtosis	1	2	3	4	5
1	BIS‐A	16.96	3.00	0.11	0.17	1	0.592***	0.506***	0.471***	0.296**
2	BIS‐M	22.21	4.70	0.37	−0.09		1	0.570***	0.501***	0.261**
3	BIS‐NP	27.54	5.45	−0.01	−0.24			1	0.408***	0.227*
4	DERS	94.16	22.94	−0.26	−0.49				1	0.493***
5	SDS	7.14	2.84	−0.17	−0.48					1

Abbreviations: BIS‐A = Barratt Impulsiveness Scale‐11—attentional impulsivity; BIS‐M = Barratt Impulsivity Scale‐11—motor impulsivity; BIS‐NP = Barratt Impulsiveness Scale‐11—non‐planning impulsivity; DERS = Difficulties in Emotion Regulation Scale; SDS = Severity Dependence Scale.

* *p* < 0.05.

** *p* < 0.01.

*** *p* < 0.001.

Multicollinearity statistics were within acceptable ranges as TI ranged from 0.69 to 0.81 and VIF ranged from 1.06 to 1.37. The mediation analyses testing the indirect effect of impulsivity on the severity of substance dependence via the emotion dysregulation are reported in Table 2. Impulsivity (BIS‐11 total score) was found to have a significant indirect effect on the severity of substance dependence trough emotion dysregulation (adjusted *R* ^2^ = 0.31, *F*_(df)_ = 10.67_(4)_, *p* < 0.001; bootstrapping [95% BCa]: total effect = 0.07 [0.02–0.12], direct effect = 0.002 [−0.05 to 0.05], indirect effect = 0.068 [0.03–0‐11]).

**TABLE 2:** Mediating effects of emotion dysregulation in the association between impulsivity and substance dependence severity.

		*B*	SE	*p*	95% BCa
Step 1	IV: Impulsivity (BIS‐11)	0.07	0.05	0.006	0.02–0.12
	DV: Severity dependence (SDS)				
Step 2	IV: Impulsivity (BIS‐11)	1.11	0.17	< 0.001	0.75–1.46
	DV: Emotion dysregulation (DERS)				
Step 3	IV: Impulsivity (BIS‐11)	0.002	0.03	0.933	−0.05 to 0.04
	M: Emotion dysregulation (DERS)	0.06	0.01	< 0.001	0.04–0.08
	DV: Severity dependence (SDS)				

Abbreviations: BIS‐11: Barratt Impulsiveness Scale‐11, DERS: Difficulties Emotion Regulation Scale; SDS: Severity Dependence Scale.

The mediation analyses testing the indirect effects of different facets of impulsivity on the severity of substance dependence via emotion dysregulation are reported in Table 3. Attentional impulsivity (BIS‐11 attentional impulsivity subscale score) was found to have a significant indirect effect on the severity of substance dependence via emotion dysregulation (adjusted *R* ^2^ = 0.31, *F*_(df)_ = 10.78_(4)_, *p* < 0.001; bootstrapping [95% BCa]: total effect = 0.27 [0.10–0.45], direct effect = 0.05 [−0.14 to 0.24], indirect effect = 0.22 [0.11–0.36]). Motor impulsivity (BIS‐11 motor impulsivity subscale score) was found to have a significant indirect effect on the severity of substance dependence trough emotion dysregulation (adjusted *R* ^2^ = 0.31, *F*_(df)_ = 10.68_(4)_, *p* < 0.001; bootstrapping [95% BCa]: total effect = 0.16 [0.08–0.24], direct effect = 0.01 [−0.11 to 0.12], indirect effect = 0.15, [0.08–0.24]). Non‐planning impulsivity (BIS‐11 non‐planning impulsivity subscale score) was found to have a significant indirect effect on the severity of substance dependence via emotion dysregulation (adjusted *R* ^2^ = 0.31, *F*_(df)_ = 10.67_(4)_, *p* < 0.001; bootstrapping [95% BCa]: total effect = 0.11 [0.01–0.21], direct effect = 0.002 [−0.09 to 0.10], indirect effect = 0.108, [0.04–0.18]).

**TABLE 3:** Mediating effects of emotion dysregulation in the association between difference facets of impulsivity and substance dependence severity.

		*B*	SE	*p*	95% BCa
Model 1: Attentional impulsivity					
Step 1	IV: Attentional impulsivity (BIS‐11)	0.27	0.09	0.003	0.10–0.45
	DV: Severity dependence (SDS)				
Step 2	IV: Attentional impulsivity (BIS‐11)	3.78	0.67	< 0.001	2.45–5.12
	DV: Emotion dysregulation (DERS)				
Step 3	IV: Attentional impulsivity (BIS‐11)	0.05	0.09	0.587	−0.13 to 0.23
	M: Emotion dysregulation (DERS)	0.06	0.01	< 0.001	0.03–0.08
	DV: Severity dependence (SDS)				
Model 2: Motor impulsivity					
Step 1	IV: Motor impulsivity (BIS‐11)	0.16	0.06	0.006	0.04–0.27
	DV: Severity dependence (SDS)				
Step 2	IV: Motor impulsivity (BIS‐11)	2.44	0.42	< 0.001	1.60–3.28
	DV: Emotion dysregulation (DERS)				
Step 3	IV: Motor impulsivity (BIS‐11)	0.008	0.06	0.886	−0.11 to 0.13
	M: Emotion dysregulation (DERS)	0.06	0.01	< 0.001	0.03–0.09
	DV: Severity dependence (SDS)				
Model 3: Non‐planning impulsivity					
Step 1	IV: Non‐planning impulsivity (BIS‐11)	0.11	0.05	0.028	0.01–0.21
	DV: Severity dependence (SDS)				
Step 2	IV: Nonplanning impulsivity (BIS‐11)	1.73	0.38	< 0.001	0.96–2.50
	DV: Emotion dysregulation (DERS)				
Step 3	IV: Non‐planning impulsivity (BIS‐11)	0.002	0.05	0.955	−0.09 to 0.10
	M: Emotion dysregulation (DERS)	0.06	0.01	< 0.001	0.04–0.08
	DV: Severity dependence (SDS)				

Abbreviations: BIS‐11: Barratt Impulsiveness Scale‐11, DERS: Difficulties Emotion Regulation Scale; SDS: Severity Dependence Scale.

## 4 Discussion

The present study aimed to explore among subjects with SUD whether impulsivity may have an indirect effect on the severity of substance dependence via the use of maladaptive emotion regulation strategies.

Comparable with previous studies (Garofalo and Velotti 2015; Jakubczyk et al. 2018; Okasha et al. 2021; Russell et al. 2017; Thrap et al. 2013), we found that substance users who scored high on the three dimensions of impulsivity (attentional, motor and non‐planning) also experienced greater levels of emotion dysregulation. Several explanations could account for these findings. As suggested by several researchers (e.g., Improvisato et al. 2024; Mansueto et al. 2022; Mansueto, Caselli, et al. 2024; Mansueto, Jarach, et al. 2024; Sheppes et al. 2015), substance users with impulsive tendencies, in response to aversive internal states, may engage in maladaptive forms of mental control such as repetitive negative thinking (e.g., worry and rumination) that (a) may magnify negative affect, leading to a cycle of intensifying dysregulated emotions and perseverative cognition (i.e., the emotional cascade) (Mansueto, Caselli, et al. 2024; Mansueto, Jarach, et al. 2024; Selby et al. 2008) or (b) can monopolize mental capacity, leaving fewer cognitive resources for adaptive emotional processing (Ehring and Watkins 2008) and increasing the likelihood of becoming stuck in a cognitive bottleneck that impairs emotion regulation (Ehring and Watkins 2008; Mansueto, Palmieri, et al. 2024; Mansueto, Caselli, et al. 2024; Mansueto, Jarach, et al. 2024). In addition, the observed association between impulsivity and emotion dysregulation in individuals with SUD can also be explained by other hypotheses, such as psychiatric comorbidity (e.g., Aslan et al. 2024; Garofalo, Velotti, Callea, et al. 2018) or shared neurocognitive processes underlying both impulsivity and emotion dysregulation (Bechara et al. 2000; Fecteau et al. 2007; Hinvest et al. 2011; Kim and Lee 2010; Krawczyk et al. 2011; Manes et al. 2002; Ochsner and Gross 2005; Ray and Zald 2012; Spinella 2004; Torresgrossa et al. 2008; Xie et al. 2011; Zeeb et al. 2010; Zeeb and Winstanley 2011).

Moreover, our findings also showed that impulsivity may have an indirect effect on the severity of substance dependence via emotion dysregulation. Substance users who score higher levels of impulsivity and who experience higher levels of emotion dysregulation could be more likely to show greater substance dependence severity. These findings suggest that substance users with impulsive tendencies may experience higher severity of substance dependence because of their problematic emotion regulation strategies. Consistent with the ‘negative reinforcement model’ (Baker et al. 2004), it could be suggested that substance users with impulsive tendencies who do not have access to adaptive emotion regulation strategies may develop a reliance on substance use as a method to control, reduce or manage own unpleasant emotional states (Baker et al. 2004; Stellern et al. 2023; Weiss et al. 2022). Likewise, according with the ‘positive reinforcement model (Cooper et al. 2016; Weiss et al. 2022) it can also be suggested that among substance users with impulsive tendencies and higher emotion dysregulation, the immediate and short‐term gratification associated with substance use may counter or distract from unpleasant emotional states that such individuals may not able to approach, tolerate or accept (Weiss et al. 2022).

Considering different facets of impulsivity, we observed that attentional impulsiveness had an indirect effect on the severity of substance dependence via emotion dysregulation. Individuals with SUD who exhibit higher attentional impulsiveness may struggle to focus and concentrate on their distressing emotions. This, in turn, may predispose them to make quick decisions concerning the use of substances as a means of shifting attention away from their own distressing emotions (Baumeister and Heatherton 1996).

Moreover, considering different facets of impulsivity, we found that motor impulsiveness had an indirect effect on the severity of substance dependence via emotion dysregulation. It could be expected that individuals with SUD with higher motor impulsiveness may experience greater difficulties in controlling their behaviour when experiencing distressing emotions. This, in turn, could increase the likelihood of engaging in rash actions, such as substance use, as an attempt to reduce or suppress the intensity of their own unwanted distressing emotions (Selby et al. 2008).

Finally, considering different facets of impulsivity we found that non‐planning impulsiveness had an indirect effect on the severity of substance dependence via emotion dysregulation. Individuals with SUD who exhibit higher non‐planning impulsiveness may be characterized by impairments in goal‐directed planning and the tendency to act without considering long‐term consequences (Stanford et al. 2009). It could be expected that, in the presence of distressing emotion, they may struggle to identify the most adaptive emotion‐regulation strategies, thus failing to access effective regulatory strategies. This, combined with their tendency to act without considering future consequences, may make substance use the most immediate way to regulate their emotions.

Overall, the results of the present study align themselves with the literature suggesting that impulsivity may have an indirect effect on health outcome via emotion dysregulation (d'Acremont and Van der Linden 2007; Estévez et al. 2024; Lazuras et al. 2019; Liau et al. 2015).

### 4.1 Clinical Implications

These preliminary findings bring us to consider their potential clinical implications. Substance users are in need of a comprehensive assessment, which should include the appraisal of impulsivity (Loree et al. 2015; Patton et al. 1995), emotion dysregulation (Gratz and Roemer 2004) and substance dependence severity (Gossop et al. 1995, 1997). Substance users could be socialized to the idea that difficulties in control one's impulsivity may be associated with higher levels of emotion dysregulation, which, in turn, may lead to greater substance dependence severity. It is plausible to expect that, among substance users with impulsive tendencies, psychological interventions promoting more adaptive emotion regulation strategies may lead to an improvement in the severity of substance dependence. Thereby, among substance users with impulsive tendencies and who concomitantly have difficulties in emotion regulation, in addition to treatments aimed to reduce impulsivity (Mungo et al. 2020), psychological interventions aimed at reducing emotion dysregulation should be delivered. Dialectical Behaviour Therapy (McMain et al. 2001) or metacognitive treatments (Mansueto, Palmieri, et al. 2024; Mansueto, Jarach, et al. 2024; Mansueto et al. 2025; Wells 2011) may be suitable clinical approaches to reduce emotion dysregulation in substance users with impulsive tendencies.

### 4.2 Limitations

The results of this study must be considered with regards to its limitations. Social desirability, self‐report biases and poor recall may have contributed to errors in self‐report measurements. The cross‐sectional design prevents us from stressing causal inferences with reasonable certainty. For instance, it is not possible to exclude that difficulties in emotion regulation may precede and pave the way for subsequent problems with impulse control, which, in turn, may lead to substance dependence (Forsén Mantilla et al. 2022; Garofalo and Velotti 2015; Waite et al. 2024). Likewise, it is not possible to exclude that substance dependence severity itself may represent a trigger, rather than an effect for impulsivity and emotions dysregulation (Jakubczyk et al. 2018). Likewise, an alternative developmental model to consider is that childhood emotion dysregulation may predispose individuals to greater impulsivity in adulthood. A further limitation is that the sample included polysubstance users, which may limit the generalizability of the mediation models across different substances. Furthermore, comorbid conditions were not considered, although these may affect the severity of dependency, either independently of, or in interaction with, impulsivity and emotion dysregulation. The present study used the DERS total score to assess emotion dysregulation, rather than its six subscales. Although this approach allows us to evaluate the impact of overall emotion dysregulation in the association between impulsivity and severity of substance use, it may prevent the estimation of the potential specific contribution of each dimension of emotion dysregulation. The decision to use the DERS total score instead of its six subscales was driven by the sample size, as analysing the subscales would have required a larger sample to obtain stable and reliable estimates of each specific dimension of emotion dysregulation. It is important also to acknowledge that previous research has raised concerns regarding the validity and reliability of the DERS Lack of Emotional Awareness subscale. Finally, dependence severity was assessed using the SDS total score (Gossop et al. 1995, 1997) rather than a DSM diagnosis (APA 2013), as it provides a continuous measure more suitable for the study aims than a categorical approach. These limitations suggest some directions for future research. Further studies with large samples, using longitudinal or experimental designs, focusing on monosubstance users and considering comorbid conditions, specific DERS dimensions and formal DSM diagnosis (APA 2013) are warranted to clarify the complex relationship between impulsivity, emotion dysregulation and severity of substance dependence.

### 4.3 Conclusion

Substance users with impulsivity tendencies may rely on maladaptive emotion regulation strategies that, in turn, may exacerbate the severity of substance dependence. From a clinical perspective, focusing solely on impulsivity in subjects with SUD may capture only a narrow part of the clinical features underlying the severity of substance dependence, potentially neglecting important features related to emotion dysregulation, which may also significantly impact it. Thereby, in addition to addressing impulsivity, targeting difficulties in emotion dysregulation (Stellern et al. 2023; Weiss et al. 2022) may represent a suitable therapeutic approach to reduce the severity of substance dependence in SUD.

## Author Contributions

**Giovanni Mansueto:** conceptualization, writing – original draft, methodology, formal analysis. **Sara Palmieri:** data curation, writing – review and editing, formal analysis. **Ana V. Nikčević:** writing – review and editing. **Gabriele Caselli:** writing – review and editing. **Marcantonio M. Spada:** writing – review and editing.

## Funding

The authors have nothing to report.

## Ethics Statement

The study was conducted in accordance with the Declaration of Helsinki, and the protocol was approved by the Ethics Committee of the Sigmund Freud University. Informed consent was obtained from all individual subjects participating in the study.

## Conflicts of Interest

The authors declare no conflicts of interest.

## Data Availability

The data that support the findings of this study are available from the corresponding author upon reasonable request.
